# Antibacterial Mode of *Eucommia ulmoides* Male Flower Extract Against *Staphylococcus aureus* and Its Application as a Natural Preservative in Cooked Beef

**DOI:** 10.3389/fmicb.2022.846622

**Published:** 2022-03-08

**Authors:** Min Xing, Shun Liu, Yaping Yu, Ling Guo, Yao Wang, Yage Feng, Peng Fei, Huaibing Kang, Md. Aslam Ali

**Affiliations:** ^1^College of Food and Bioengineering, Henan University of Science and Technology, Luoyang, China; ^2^Henan International Joint Laboratory of Food Green Processing and Quality Safety Control, Henan University of Science and Technology, Luoyang, China; ^3^School of Zhang Zhongjing Health Care and Food, Nanyang Institute of Technology, Nanyang, China; ^4^Key Lab of Dairy Science, Ministry of Education, College of Food Science, Northeast Agricultural University, Harbin, China; ^5^Department of Agro-Processing, Bangabandhu Sheikh Mujibur Rahman Agricultural University, Gazipur, Bangladesh

**Keywords:** *Eucommia ulmoides* male flower extract, *Staphylococcus aureus*, antibacterial mode, natural preservative, cooked beef

## Abstract

The research was conducted to elucidate the antibacterial performance and mode of action of *Eucommia ulmoides* male flower extract (EUMFE) against *Staphylococcus aureus* and its application as a natural preservative in cooked beef. The antibacterial activity was evaluated by determining the diameter of inhibition zone (DIZ), minimum inhibitory concentration (MIC), and minimum bactericide concentration (MBC). The changes in membrane potential, contents of bacterial DNA and protein, integrity and permeability of the cell membrane, and cell morphology were analyzed to reveal the possible mode of action. The effect of EUMFE on the counts of *S. aureus*, pH, color, total volatile basic nitrogen (TVB-N), and thiobarbituric acid reactive substances (TBARS) of the cooked beef stored at 4°C for 9 days were studied. The results showed that the DIZ, MIC, and MBC of EUMFE against *S. aureus* were 12.58 ± 0.23 mm, 40 mg/mL, and 80 mg/mL, respectively. The mode of action of EUMFE against *S. aureus* included hyperpolarization of cell membrane, decrease in bacterial DNA and protein contents, destruction of cell membrane integrity, increase in cell membrane permeability, and damage of cell morphology. After treatments with EUMFE, the growth of *S. aureus* and lipid oxidation in cooked beef were significantly inhibited (*P* < 0.05). The pH and TVB-N values of cooked beef treated with EUMFE were significantly reduced as compared to control group (*P* < 0.05). The color of cooked beef samples containing EUMFE showed decreased L* and b* values, and increased a* and ΔE* values. Therefore, our findings showed that EUMFE had a good antibacterial effect on *S. aureus*, and provided a theoretical basis for the application of EUMFE as a natural preservative in the preservation of cooked beef.

## Introduction

*Staphylococcus aureus*, a facultative anaerobe and gram-positive food-borne pathogen, is abundantly distributed in water, air, dust, and animal feces. The Staphylococcal enterotoxins produced by it are associated with food spoilage and food poisoning (Wang et al., 2018; Liang et al., 2020). In addition, *S. aureus* can cause human infectious diseases including suppurative skin infections, endocarditis, pneumonia, and bacteremia, with a higher mortality rate (Wang et al., 2020).

In recent years, the contamination of cooked meat products with *S. aureus* has been concerned as severe health problems faced by food industry and consumers (Ifesan et al., 2009). Chemical preservatives have commonly been used to inhibit *S. aureus* growth as well as prolong the shelf life of food products due to their lower cost and better effects (Ghabraie et al., 2016). However, some studies have suggested that the long-term use and consequent residual toxicity of chemical preservatives has carcinogenic and teratogenic effects which pose a significant risk to consumers’ health (Diao et al., 2018). Therefore, more attention has been paid to natural preservatives for their green, safe, and non-polluting characteristics.

*Eucommia ulmoides* Oliver, belonging to monotypic family Eucommiaceae, is a woody perennial dioecious plant native to China and its male flower has favorable prospects (Ding et al., 2015). The male flowers of *E. ulmoides* are available in relatively large yields and are easy to harvest (Ding et al., 2020). Moreover, *E. ulmoides* male flower is abundant in phytonutrients and various biologically active substances including phenols, flavonoids, chlorogenic acid, and aucubin, which possess stronger antioxidant, anti-inflammatory, antihypertensive, and analgesic effects (Xing et al., 2019). For decades, *E. ulmoides* male flower has been widely used as healthcare tea and herbal medicine in China (Dong et al., 2011). However, the research on the antibacterial effect of *E. ulmoides* male flower against food-borne pathogens has not yet been progressed which limits its application in food preservation to a certain extent.

Therefore, the present study aimed to assess the antibacterial activity and mode of action of *E. ulmoides* male flower extract (EUMFE) against *S. aureus* in terms of changes in membrane potential, contents of bacterial DNA and protein, integrity and permeability of cell membrane, and cell morphology. Furthermore, the application feasibility of the EUMFE as a natural preservative in cooked beef was also investigated.

## Materials and Methods

### Main Reagents

Propidium iodide (PI) was purchased from Beijing Solarbio Technology Co., Ltd. (Beijing, China). All media were obtained from Shanghai SIG Biotechnology Co., Ltd. (Shanghai, China). All other chemicals and reagents used in this study were of analytical grade and provided by Xi’an Jinyuan Biotechnology Co., Ltd. (Xi’an, China).

### Bacterial Strain and Culture Condition

*Staphylococcus aureus* (ATCC 25923) was obtained from the American Type Culture Collection (ATCC, Manassas, VA, United States) and stored at −80°C in Luria-Bertani (LB) broth with 20% glycerol (v/v). Before each experiment, the strain was re-cultured in LB broth at 37°C for 20 h and streaked onto Tryptone soy agar (TSA) plate followed by incubating at 37°C for 24 h to get individual colonies. A typical colony was transferred into LB broth followed by incubating at 37°C for 24 h for further use.

### Preparation of *Eucommia ulmoides* Male Flower Extract

The *E. ulmoides* male flower were first crushed to a fine powder to pass 60-mesh sieve, and then extracted by ultrasound assistance for 2 h using 70% ethanol (v/v) as an extraction solvent at a ratio of 1:30 (g/mL). The extracted suspension was filtered, and two extractions for the filter residues were performed by following the same procedure. The collected filtrate was evaporated at 40°C in a rotary-evaporator (Shanghai Kexiao Scientific Instrument Co., Ltd., Shanghai, China) to remove ethanol and concentrated until a “paste like form” was obtained. Finally, the paste was freeze-dried (−80°C) for 24 h to obtain EUMFE powder. The images of *E. ulmoides* male flower and EUMFE powders were shown in Supplementary Figure 1. The major chemical components in EUMFE were tabulated in Supplementary Table 1.

### Determination of Diameter of Inhibition Zone

The diameter of inhibition zone (DIZ) of EUMFE against *S. aureus* was determined using the Oxford cup method as described by Chang et al. (2021). TSA plates were prepared by pouring 20 mL of TSA medium onto sterile glass petri dishes. 100 μL of *S. aureus* suspension (approximately 10^7^ CFU/mL) was uniformly spread on TSA plates. Three sterile oxford cups (8 mm in diameter) were placed on the surface of each TSA plate followed by addition of 200 μL EUMFE (160 mg/mL). After incubation at 37°C for 24 h, the DIZ was measured with a vernier caliper and recorded in mm.

### Determination of Minimum Inhibitory Concentration and Minimum Bactericide Concentration

The minimum inhibitory concentration (MIC) and minimum bactericide concentration (MBC) of EUMFE against *S. aureus* was determined by agar dilution method (Fei et al., 2020). In brief, EUMFE was thoroughly mixed with sterilized TSA medium (at about 50°C) in a 24-well plate to obtain the final concentrations of 5, 10, 20, 40, 80, and 160 mg/mL. After solidifying, each well was inoculated with 2 μL of bacterial suspension of *S. aureus* (10^6^ CFU/mL) and incubated at 37°C for 24 h. The MIC was defined as the lowest concentration of EUMFE with no visual bacterial growth. Further, after treatments with ≥ 1 MIC of EUMFE for 30 min, 100 μL of *S. aureus* suspension (10^6^ CFU/mL) was spread on TSA plate and incubated at 37°C for 24 h. The MBC was interpreted as the lowest concentration of EUMFE inhibiting growth of colonies of *S. aureus*. As a validation experiment, the growth curves of *S. aureus* treated with different concentrations (0 MIC, 1/4 MIC, 1/2 MIC, 1 MIC, and 2 MIC) of EUMFE were measured by the method of Shi et al. (2016).

### Measurement of Membrane Potential

The effect of EUMFE on the membrane potential of *S. aureus* was analyzed as described by Guo et al. (2019a). Briefly, 0.5 μL of bis-(1,3-dibutylbarbituric acid) trimethine oxonol [DiBAC_4_(3); Beijing Solarbio Science and Technology Co. Ltd., Beijing, China] was added to a 96-well microtiter plate containing 125 μL of *S. aureus* suspension (10^7^ CFU/mL) and cultured at 37°C for 24 h, followed by addition of EUMFE at final concentrations of 0 MIC (control), 1 MIC, and 2 MIC. The fluorescence intensities of the experimental samples were measured by a multifunctional microplate reader (Nanjing Junwei Biotechnology Co. Ltd., Jiangsu, China) with excitation/emission wavelengths of 492/515 nm at 37°C.

### Agarose Gel Electrophoresis for DNA Fragmentation

The effect of EUMFE on the genomic DNA of *S. aureus* was analyzed using agarose gel electrophoresis according to the report of Guo et al. (2020). Approximately 10^8^ CFU/mL of *S. aureus* culture were treated with 0 MIC (control), 1 MIC and 2 MIC of EUMFE at 37°C for 1, 3, 5, 7, and 9 h. The genomic DNA was extracted using a bacterial genomic DNA extraction kit (Tiangen Biotechnology Co., Ltd., Beijing, China). After electrophoresis with 1.5% agarose gel at 100 V for 30 min, the gels were stained with 10 mg/mL of ethidium bromide for 15 min and visualized through the gel imaging system (Bio-Rad Laboratories, Hercules, CA, United States).

### Sodium Dodecyl Sulfate-Polyacrylamide Gel Electrophoresis

Sodium dodecyl sulfate-polyacrylamide gel electrophoresis (SDS-PAGE) can separate the protein into several bands in electrophoretic gel according to the different molecular weight and charge of the protein, and the bands with high protein content are deep. SDS-PAGE analysis of bacterial proteins was performed following the procedure as described by Fei et al. (2019). In brief, *S. aureus* culture (10^7^ CFU/mL) was treated with 0 MIC (control), 1 MIC, and 2 MIC of EUMFE for 1, 3, 5, 7, and 9 h. The cell pellets were prepared by centrifugation at 8000 × g for 10 min at 4°C, then resuspended in normal saline followed by mixing with SDS-PAGE loading buffer. After heating at 95°C for 10 min, the samples were analyzed by SDS-PAGE with 5% stacking gel and 15% separating gel, and the protein bands were stained with Coomassie brilliant blue R-250 (Beijing Zhongsheng Ruitai Technology Co. Ltd., Beijing, China). Finally, the image was taken with the HP scanner (HP 1000, Hewlett-Packard Co. Ltd., Beijing, China).

### Fluorescence Microscope

The fluorescence microscope observation was used to evaluate the changes in membrane integrity of *S. aureus* according to the previously reported method (Han et al., 2021). Briefly, after treatments with 0 MIC (control), 1 MIC, and 2 MIC of EUMFE at 37°C for 3 h, the *S. aureus* culture (10^8^ CFU/mL) was harvested by centrifugation at 8000 × *g* for 10 min and then washed three times followed by re-suspension in 0.01 M sterilized phosphate buffered solution (PBS). The bacterial suspension was incubated with PI dye (2 μg/mL) at room temperature (25 ± 2°C) in the dark for 30 min. Finally, 10 μL from aforementioned culture was observed and imaged under the fluorescence microscopy (Hunan Andao Technology Co. Ltd, Hunan, China) using excitation and emission wavelengths of 536 and 617 nm respectively.

### Flow Cytometry

The effect of EUMFE on membrane permeability of *S. aureus* was assessed by the procedure as described by Kang et al. (2020). *S. aureus* suspension (10^8^ CFU/mL) was treated with 0 MIC (control), 1 MIC and 2 MIC of EUMFE and incubated at 37°C for 3 h followed by centrifugation at 5000 × *g* for 10 min. The collected cells were stained with 10 μg/mL PI dye for 30 min at room temperature (25 ± 2°C) in the dark, and detected by a flow cytometer (Shenzhen Mindray Bio-Medical Electronics Co. Ltd., Guangdong, China).

### Observation of Morphological Changes of *Staphylococcus aureus*

According to the report of Wang et al. (2018), the morphological changes of *S. aureus* cells treated with different concentrations of EUMFE were observed under Scanning Electron Microscopy (SEM) and Transmission Electron Microscopy (TEM). After treatments with EUMFE of 0 MIC (control), 1 MIC, and 2 MIC for 3 h, the suspensions of *S. aureus* (10^7^ CFU/mL) were centrifuged at 4000 × *g* for 10 min at 4°C to get the cell pellets, which were washed three times with 0.01 M PBS and fixed in 2.5% glutaraldehyde at 4°C for 12 h. The cultures were dehydrated with a series of dilutions of ethanol including 30, 50, 70, 90, and 100% ethanol for 15 min. Finally, the experimental samples were dried in freeze dryer, coated with gold and subjected to observations under the SEM (TM3030, Hitachi, Tokyo, Japan).

The pretreatment of *S. aureus* culture for TEM was performed by following the similar procedure as mentioned above for SEM. The prefixed cells were fixed up by 1% osmic acid for 2 h and washed three times with PBS. After dehydrating with different concentrations of ethanol, the cells were embedded by Epon Lx-112 (Ladd Research, Williston, VT, United States) and double-stained with uranyl acetate and lead citrate. Eventually, all samples were observed under the TEM (Hitachi, Tokyo, Japan).

### Preparation of Cooked Beef Samples

The cooked beef samples were prepared according to the procedure as reported by Gong et al. (2021). Fresh beef purchased from the local supermarket was chopped into small pieces keeping a weight around 1.5 g and sterilized at 121°C for 15 min to obtain sterile cooked beef samples. These sterile samples were dipped into EUMFE solutions with the concentrations 0 MIC (control), 1 MIC, and 2 MIC for 10 s and placed in petri dishes for 30 min followed by packaging in sterile polythene bags. Finally, all samples were stored at 4°C for further analyses.

### Microbiological Analysis

Cooked beef samples were inoculated with *S. aureus* to approximately 10^3^ CFU/g and stored at 4°C for 0, 3, 6, and 9 days. The inoculated samples (1 g) were thoroughly mixed with 9 mL of sterile normal saline (NS), and a 10-fold serial dilution was prepared. 100 μL from each dilution was evenly spread onto TSA plate individually and incubated at 37°C for 24 h (Guo et al., 2020).

### pH Measurement

The pH values of cooked beef samples were determined using the method of Carpenter et al. (2007). On days 0, 3, 6, and 9, cooked beef samples (1 g) were homogenized in 9 mL of distilled water for 1 min. The pH of the homogenate was measured using a portable pH-meter (Radiometer, Copenhagen, Denmark).

### Color Measurement

According the report of Pissaia et al. (2015), a CR-300 Chroma Meter (Minolta Co., Osaka, Japan) was used to measure the color parameters including L*(lightness), a*(redness), and b*(yellowness) of cooked beef samples after 0, 3, 6, and 9 days of storage at 4°C. In addition, the total color difference (ΔE*) was calculated by the following formula: ΔE* = [(ΔL*)^2^ + (Δa*)^2^ + (Δb*)^2^]^1/2^.

Where ΔL*, Δa*, and Δb* are the difference of L*, a*, and b* of the samples between control and the EUMFE treatments.

### Determination of Total Volatile Basic Nitrogen

The total volatile basic nitrogen (TVB-N) value of cooked beef samples was measured according to the method as mentioned by Zhang et al. (2011). On days 0, 3, 6, and 9, each sample (1 g) was homogenized in 10 mL distilled water for 30 min and then filtered. 5 mL of filtrate and 5 mL of MgO solution was distilled through Kjeldahl Apparatus (Shanghai Yihong Analytical Instrument Co. Ltd., Shanghai, China). The distillate was collected in a flask containing 10 mL of aqueous solution of boric acid (20 g/L) and a mixed indicator prepared by dissolving equal weight (0.1 g) of methyl red and methylene blue in 100 mL ethanol (w/v). The flask’s content was then titrated against 0.01 mol/L hydrochloric acid. The TVB-N value was determined according to the consumption of hydrochloric acid and expressed as mg per 100 g cooked beef.

### Determination of Thiobarbituric Acid Reactive Substances

The thiobarbituric acid reactive substances (TBARS) value of cooked beef sample was determined by following the method as stated by Yarnpakdee et al. (2012). Every 3 days during storage, the cooked beef sample (1 g) was mixed with 5 mL of thiobarbituric acid (TBA) reagent consisting of 0.375% thiobarbituric acid, 15% trichloroacetic acid, and 0.25 M HCl. The mixture was heated in a boiling water bath for 10 min to develop a pink color. After cooling with running tap water, the mixture was centrifuged at 3600 × *g* for 20 min to obtain supernatant. Finally, the absorbance of obtained supernatant was measured at a wavelength 532 nm. 1,1,3,3-tetramethox-ypropane was used to build the standard curve, and the TBARS value was calculated and expressed as mg malonaldehyde per kg of sample.

### Statistical Analysis

All experiments were conducted in triplicate and results were expressed as mean ± standard deviation (SD). The data were analyzed using analysis of variance (ANOVA) in the SPSS 20.0 software. The difference was considered statistically significant when *P*-values less than 0.05.

## Results

### Diameter of Inhibition Zone, Minimum Inhibitory Concentration, and Minimum Bactericide Concentration of *Eucommia ulmoides* Male Flower Extract Against *Staphylococcus aureus*

The results showed that the mean DIZ value of EUMFE against *S. aureus* was 12.58 ± 0.23 mm. Meanwhile, the MIC and MBC of EUMFE against *S. aureus* were 40 and 80 mg/mL, respectively. In addition, the growth curve of *S. aureus* under action of 1 MIC of EUMFE was almost stagnant (Supplementary Figure 2), which indicated that the result of MIC value was valid.

### Changes in Membrane Potential

**FIGURE 1:**
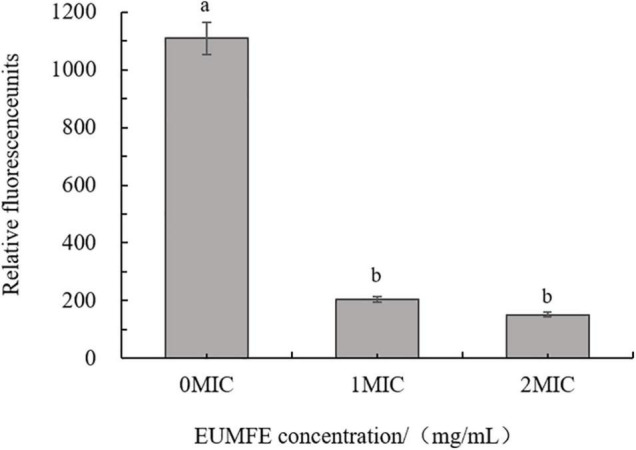
Effect of *Eucommia ulmoides* male flower extract (EUMFE) on the membrane potential of *Staphylococcus aureus*. Each bar represents the mean ± SD of three independent experiments. Different lowercase letters indicate the significant difference at *P* < 0.05.

As shown in Figure 1, the fluorescence intensities of *S. aureus* cells treated with 1 and 2 MIC of EUMFE were significantly reduced as compared to the control group (0 MIC) (*P* < 0.05), which indicated that treatment with EUMFE resulted in hyperpolarization of *S. aureus* cell membrane. Besides, the results obtained in this study (Figure 1) showed no significant difference in fluorescence intensity between the cells treated with 1 and 2 MIC of EUMFE.

### DNA Fragmentation Analysis

**FIGURE 2:**
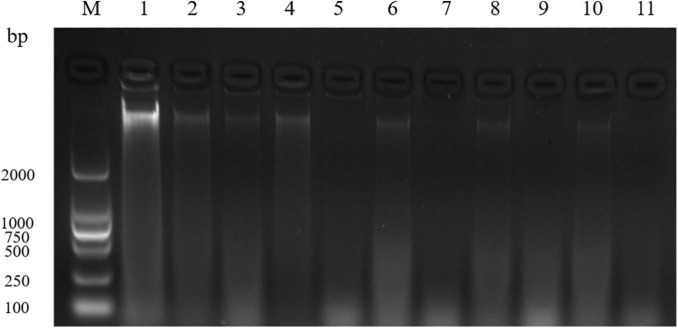
Agarose gel electrophoretogram of DNA in *Staphylococcus aureus* treated with 1 and 2 MIC of *Eucommia ulmoides* male flower extract (EUMFE). Lane M: marker; Lane 1: control; Lane 2, 4, 6, 8, and 10: treatment with 1 MIC of EUMFE for 1, 3, 5, 7, and 9 h, respectively. Lane 3, 5, 7, 9, and 11: treatment with 2 MIC of EUMFE for 1, 3, 5, 7, and 9 h, respectively.

Figure 2 showed that the DNA bands of *S. aureus* cells treated with 1 and 2 MIC of EUMFE in genomic DNA electrophoretogram were faded in comparison with untreated cells. The bacterial DNA bands got much fainter with increase in treatment time and concentration, and almost disappeared after 9 h of treatment with 2 MIC of EUMFE.

### Sodium Dodecyl Sulfate-Polyacrylamide Gel Electrophoresis Analysis

**FIGURE 3:**
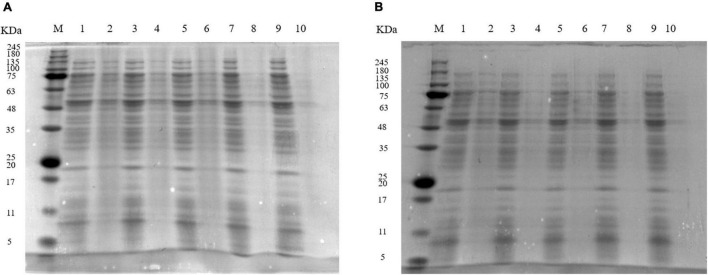
Sodium dodecyl sulfate-polyacrylamide gel electrophoresis (SDS-PAGE) profiles of proteins in *Staphylococcus aureus* treated with **(A)** 1 MIC and **(B)** 2 MIC of *Eucommia ulmoides* male flower extract (EUMFE). Lane M: marker; Lane 1, 3, 5, 7, and 9: control; lane 2, 4, 6, 8, and 10: treatment with 1 MIC or 2 MIC of EUMFE for 3, 6, 9, 12 h, respectively.

The SDS-PAGE profiles (Figure 3) showed that protein bands of *S. aureus* cells treated with 1 MIC and 2 MIC of EUMFE got weaker as compared to those of control group (0 MIC), and the degree of protein bands weakening became more pronounced as the treatment time and concentration increased. Furthermore, the protein bands of *S. aureus* completely disappeared after treatments with 1 and 2 MIC of EUMFE for 9 h.

### Fluorescence Microscope Observation

**FIGURE 4:**
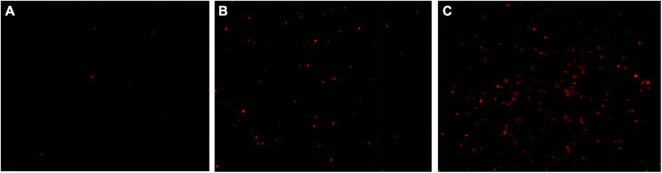
Fluorescence microscope images of *Staphylococcus aureus* cells stained with propidium iodide (PI) after treatments with *Eucommia ulmoides* male flower extract (EUMFE) for 3 h. **(A)** Untreated cells; **(B)** cells treated with EUMFE at 1 MIC; **(C)** cells treated with EUMFE at 2 MIC.

The results of fluorescence microscope images showed that only very few cells were red for the control group (0 MIC) (Figure 4A). After treatments with 1 MIC of EUMFE for 3 h, a little red fluorescence in the field of vision was observed (Figure 4B). On the other hand, the red fluorescence appeared increasingly against an increase in concentration of EUMFE (Figure 4C).

### Flow Cytometer Analysis

**FIGURE 5:**
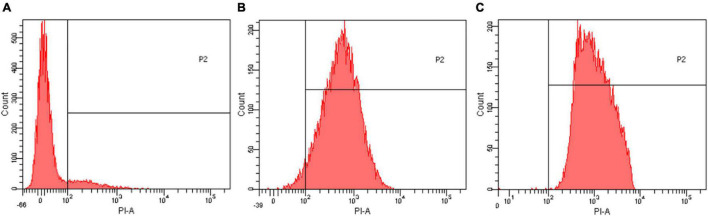
Flow cytometric histograms of *Staphylococcus aureus* stained with propidium iodide (PI) after treatments with *Eucommia ulmoides* male flower extract (EUMFE) for 3 h. **(A)** Untreated cells; **(B)** cells treated with EUMFE at 1 MIC; **(C)** cells treated with EUMFE at 2 MIC.

The effect of EUMFE on membrane permeability of *S. aureus* was shown in Figure 5. Compared to the control group (0 MIC) (Figure 5A), the percentage of *S. aureus* cells stained with PI (in the area to the right of the vertical line) was significantly increased by following the exposure to 1 and 2 MIC of EUMFE for 3 h (Figures 5B,C). The results obtained in this study (Figure 5) indicated that EUMFE treatment increased the cell membrane permeability of *S. aureus*.

### Scanning Electron Microscopy and Transmission Electron Microscopy Observations

**FIGURE 6:**
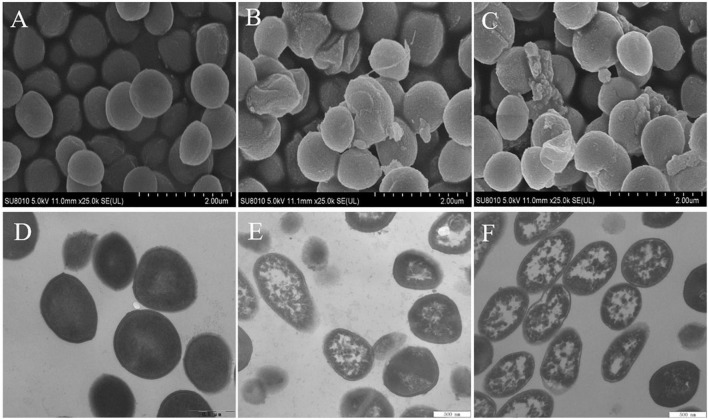
Scanning Electron Microscopy (SEM) and Transmission Electron Microscopy (TEM) images of *Staphylococcus aureus* after treatments with *Eucommia ulmoides* male flower extract (EUMFE) for 3 h. Panels **(A–C)** are SEM images of the cells untreated, treated with 1 and 2 MIC of EUMFE, respectively; Panels **(D–F)** are TEM images of the untreated, treated with 1 and 2 MIC of EUMFE, respectively.

The morphological changes of *S. aureus* cells after treatments with EUMFE for 3 h were observed by SEM and TEM (Figure 6). Untreated *S. aureus* cells remained the normal cell morphology of typical spherical shape, intact and smooth surfaces as well as homogeneous electron density in the cytoplasm (Figures 6A,D). In contrast, *S. aureus* treated with 1 (Figures 6B,E) and 2 MIC (Figures 6C,F) of EUMFE exhibited severe morphological damages including the surface depression, cell distortion, and leakage of intracellular materials. Moreover, the damages were obviously enhanced with increasing EUMFE concentration.

### Application in Cooked Beef

**FIGURE 7:**
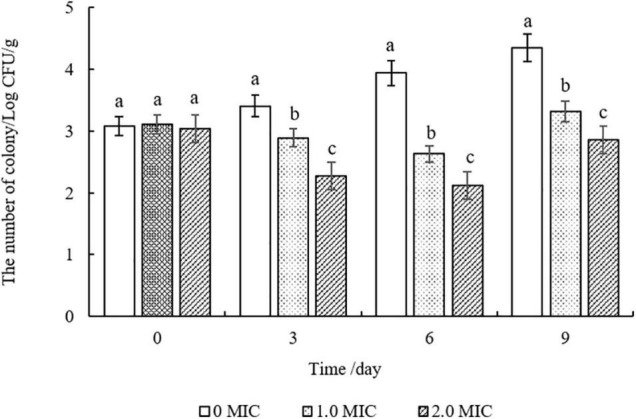
Inhibitory effect of *Eucommia ulmoides* male flower extract (EUMFE) on *Staphylococcus aureus* in cooked beef samples during storage at 4°C. Each bar represents the mean ± SD of three independent experiments. Different lowercase letters indicate the significant difference at *P* < 0.05.

As shown in Figure 7, compared to the control group (0 MIC), the number of *S. aureus* strains in cooked beef after treatments with EUMFE were reduced significantly (*P* < 0.05) on days 3, 6, and 9 of storage, and decreased significantly (*P* < 0.05) with an increase in concentration of EUMFE in the treatment medium. In addition, the number of *S. aureus* treated with EUMFE was slightly higher than that of initial populations on day 9.

### pH Changes

The changes in pH values of cooked beef samples during storage were shown in Table 1. After treatments with different concentrations of EUMFE, significantly lower pH values were measured for cooked beef than that of control group (0 MIC) (*P* < 0.05). However, no significant changes in pH value in cooked beef samples, treated with EUMFE of similar concentration, were observed by 9 days of storage (*P* > 0.05).

**TABLE 1:** The pH of cooked beef after treatments with *Eucommia ulmoides* male flower extract (EUMFE) of different concentrations during storage for 9 days.

Storage time (day)	Treatments		
	
	Control (0 MIC)	1 MIC	2 MIC
0	5.86 ± 0.02*^aA^*	5.74 ± 0.02*^bA^*	5.69 ± 0.01*^cA^*
3	5.86 ± 0.01*^aA^*	5.72 ± 0.01*^bA^*	5.67 ± 0.02*^cA^*
6	5.87 ± 0.02*^aA^*	5.72 ± 0.01*^bA^*	5.68 ± 0.02*^cA^*
9	5.88 ± 0.01*^aA^*	5.73 ± 0.01*^bA^*	5.67 ± 0.01*^cA^*

*Values were expressed as mean ± SD. The similar superscripts in a single column indicates no significant difference (P < 0.05). Different lowercase superscripts (a–c) in a row indicate significant differences between treatments. The same uppercase letters (A) in a column indicate no significant difference during storage time in each treatment (P < 0.05).*

### Color Changes

As shown in Table 2, compared to the control group (0 MIC), the cooked beef samples treated with EUMFE showed significantly lower L* and b* values, and higher a* and ΔE* values (*P* < 0.05). With an extension of storage time, there was significant decreases in L* values and increases in b* values (*P* < 0.05) of all samples. In addition, the a* values of control samples (0 MIC) significantly decreased with an increase in storage time (*P* < 0.05) while no significant differences in a* values were found in cooked beef treated with EUMFE by first 6 days of storage (*P* > 0.05).

**TABLE 2:** The color of cooked beef treated with different concentrations of *Eucommia ulmoides* male flower extract (EUMFE) during storage for 9 days.

Storage time (day)	Treatments		
	
	Control (0 MIC)	1 MIC	2 MIC
L*-value			
0	52.54 ± 0.21*^aA^*	44.23 ± 0.23*^bA^*	37.93 ± 0.07*^cA^*
3	51.28 ± 0.12*^aB^*	43.52 ± 0.07*^bB^*	36.88 ± 0.05*^cB^*
6	48.94 ± 0.32*^aC^*	42.45 ± 0.14*^bC^*	36.42 ± 0.04*^cB^*
9	48.16 ± 0.22*^aD^*	42.08 ± 0.29*^bC^*	36.27 ± 0.10*^cB^*
a*-value			
0	4.57 ± 0.09*^cA^*	10.83 ± 0.11*^bA^*	13.62 ± 0.32*^aA^*
3	4.13 ± 0.08*^cB^*	10.72 ± 0.08*^bA^*	13.49 ± 0.05*^aA^*
6	3.78 ± 0.04*^cC^*	10.67 ± 0.12*^bA^*	13.38 ± 0.04*^aA^*
9	3.02 ± 0.14*^cD^*	10.28 ± 0.10*^bB^*	13.28 ± 0.09*^aA^*
b*-value			
0	22.34 ± 0.19*^aC^*	16.04 ± 0.21*^bB^*	12.49 ± 0.18*^cB^*
3	22.76 ± 0.17*^aC^*	16.41 ± 0.11*^bA^*	12.68 ± 0.05*^cA^*
6	24.35 ± 0.26*^aB^*	16.12 ± 0.07*^bB^*	12.71 ± 0.03*^cA^*
9	25.23 ± 0.11*^aA^*	16.56 ± 0.09*^bA^*	12.77 ± 0.02*^cA^*
ΔE*-value			
0		12.16 ± 0.12*^bB^*	19.82 ± 0.12*^aA^*
3		12.01 ± 0.27*^bB^*	19.91 ± 0.18*^aA^*
6		12.58 ± 0.08*^bA^*	19.63 ± 0.36*^aB^*
9		12.84 ± 0.15*^bA^*	20.05 ± 0.14*^aA^*

*Values were expressed as means of three independent replicates ± SD.*

Values followed by different lowercase superscripts (a–c) within the same row are significantly different (P < 0.05). Values followed by different uppercase superscripts (A–D) within the same column are significantly different (P < 0.05). L*, a*, b*, and ΔE* represent brightness, redness, yellowness and total color difference respectively, which is a fixed representation.

### Changes in Total Volatile Basic Nitrogen and Thiobarbituric Acid Reactive Substances of Cooked Beef

**FIGURE 8:**
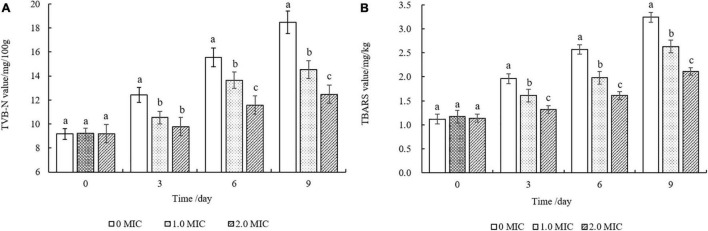
Changes in TVB-N **(A)** and TBARS **(B)** values of cooked beef treated with different concentrations of *Eucommia ulmoides* male flower extract (EUMFE) during storage at 4°C. Each bar represents the mean ± SD of three independent experiments. Different lowercase letters indicate the significant difference at *P* < 0.05.

Figure 8 showed that the TVB-N and TBARS values of all cooked beef samples continuously increased during the storage of 9 days. The TVB-N values of the samples treated with EUMFE were significantly lower than those for untreated samples from the third day (*P* < 0.05), and no significant difference (*P* > 0.05) was found in TVB-N values between 1 and 2 MIC treated samples by 3 days (Figure 8A).

In addition, cooked beef samples treated with 1 and 2 MIC of EUMFE exhibited significantly lower lipid oxidation in comparison with control group (0 MIC) after 3 days of storage (*P* < 0.05) (Figure 8B).

## Discussion

Plant extracts and their biologically active components are considered as potential natural antimicrobials and preservatives because of their ideal antibacterial roles against food-borne pathogens (Liu et al., 2020). In our study, the MIC of EUMFE against *S. aureus* was 40 mg/mL, which is higher than those of dihydromyricetin (1.25 mg/mL), vine tea extract (6.3 mg/mL), and aqueous garlic extract (24 mg/mL) (Lee S.Y. et al., 2015; Liang et al., 2020). In the context of this study, it is obvious that EUMFE is significantly effective in inhibition of *S. aureus*, and exhibits a stronger antibacterial effect against *S. aureus* as compared to *Amaranthus tricolor* crude extract (ATCE) (80 mg/mL) (Guo et al., 2020). Of course, it is not enough to only study the antibacterial effect of EUMFE on *S. aureus*. In the future, we will continue to study the antibacterial effect of EUMFE on other food-borne pathogens to determine whether EUMFE has a broad-spectrum antibacterial effect.

Membrane potential refers to the potential differences between two sides of a cell membrane during the resting state of the cells, as it closely relates to antibiotic uptake and bactericidal actions by the cells (Shi et al., 2016). Our results showed that EUMFE caused hyperpolarization of cell membranes of *S. aureus* which might be occurred primarily in response to pH change and K^+^ efflux (Bot and Prodan, 2009). Similarly, cell membrane hyperpolarization was found when *S. aureus* exposed to *2R*,*3R*-dihydromyricetin, shikimic acid, and chlorogenic acid (Li et al., 2014; Bai et al., 2015; Wu et al., 2017). As a contrast, cell membrane depolarization associated with migration of Na^+^ was observed in *S. aureus* while treated with olive oil polyphenols extract (OOPE) and fermented *Inula britannica* extract (Bae et al., 2019; Guo et al., 2019b).

DNA is vital for survival of bacterial cells because it is an important genetic material that control bacterial growth, development, and inheritance (Kang et al., 2020). In the present study, after treatments with EUMFE, the DNA fragmentation appeared in *S. aureus* cells, which is consistent with the changes in bacterial DNA in the inhibition of ATCE against *S. aureus* (Guo et al., 2020). Similarly, a study of Liu et al. (2011) indicated cajanol inhibited the growth of *Escherichia coli* and *S. aureus* through DNA cleavage. Wu et al. (2017) reported that *2R*,*3R*-dihydromyricetin can cause the nucleic acid leakage from *S. aureus* through a destruction of cell membrane and interact with genomic DNA by the groove binding mode, and thereby reduce the bacterial DNA contents.

Proteins are essential biological macromolecules of bacterial cells and they are closely related to the bacterial metabolism and physiological functions (Fei et al., 2019). SDS-PAGE analysis results indicated that after treatments with EUMFE, the protein levels in *S. aureus* were obviously reduced. Similar findings have been reported in the action process of sugar beet molasses polyphenols on *S. aureus, E. coli*, *Listeria monocytogenes*, and *Salmonella typhimurium* (Chen et al., 2017). In addition, Song et al. (2020) suggested that mandarin essential oil treatment affects the protein synthesis and related gene expression in *S. aureus*. Yoo et al. (2021) believed that the reduction in protein contents of *S. aureus* cells treated with clove oil might be associated with the protein leakage caused by an increase of cell membrane permeability.

Propidium iodide is a DNA stain reagent that can penetrate membranes of damaged cells and embed in double-stranded DNA to exhibit red fluorescence. Therefore, PI staining could frequently be used to assess the extent of damage to the cell membrane (Yang et al., 2020). In this study, after a treatment with EUMFE, the percentage of staining of *S. aureus* cells with PI as well as red fluorescence under fluorescence microscope increased significantly, which revealed that EUMFE induced cell membrane damage in *S. aureus*, destroyed membrane integrity and increased membrane permeability. Han et al. (2021) found the similar results where the limonene significantly destroyed cell membrane integrity of *S. aureus*. Besides, Shi et al. (2017) reported that severe membrane damage of *S. aureus* was induced while treated with a combination of nisin and *p*-Anisaldehyde for 3 h.

Scanning electron microscopy and transmission electron microscopy observations can reflect the changes in cell morphology and ultrastructure of the tested bacteria caused by natural products on the whole. Kang et al. (2019) reported that peppermint essential oil irreversibly damages the cell membranes of *S. aureus*, causing leakage of intracellular substances. Zhang et al. (2016) indicated that the antibacterial effect of cinnamon essential oil against *S. aureus* is achieved by a direct change in the structure of cell wall and cell membrane. In the current study, severe cell morphological damage and cytoplasmic content leakage were observed in *S. aureus* while exposed to EUMFE for 3 h. These findings further support the other results, obtained in this study, including cell membrane hyperpolarization, DNA fragmentation, bacterial protein reduction and membrane damage in *S. aureus* cells being induced by EUMFE treatment. Therefore, we speculate that EUMFE damages the membrane of *S. aureus* cell, reducing the content of intracellular materials and eventually resulting in cell death.

Although the individually cooked meat products are commercially sterile, there is a risk of possible contamination by food-borne pathogens during transportation, storage, and other processes. Meanwhile, cooked meat produced in small scale industries are usually sold and stored in unhygienic even poor conditions being more likely to be contaminated by food-borne pathogens (Guo et al., 2020). Previous studies reported that natural antibacterial products can inhibit the growth of food-borne pathogens in cooked meat products, preventing them from being contaminated by microorganisms. For instance, Gong et al. (2021) found a significant reduction in *S. aureus* count in cooked pork and beef treated with cranberry anthocyanin. Apostolidis et al. (2008) reported that oregano, cranberry, and sodium lactate combination possessed obvious antibacterial effect on *L. monocytogenes* in cooked beef. Similarly, the addition of EUMFE reduced the number of *S. aureus* in cooked beef, indicating that EUMFE could effectively inhibit the growth of *S. aureus* in cooked beef. Besides, because it is the preliminary study to evaluate the behavior of EUMFE inside a food matrix, a small sample size (1 g) was used in this research. In future studies, commercial hunk of cooked beef will be used as samples to evaluate the potential of EUMFE as a natural preservative.

The decrease of pH due to the addition of natural antibacterials in cooked meat products is helpful to inhibit the growth of undesirable bacteria and extend the shelf life of products (Ozaki et al., 2020). According to the report of Lucas and Were (2009), who found the addition of lyophilized pomegranate juice could effectively reduce the pH of cooked ground beef during storage. Ahn et al. (2007) reported that the efficacy of the pycnogenol treatment for inhibiting microbial growth in cooked beef attributes to the lower pH of cooked meat by gallic acid, vanillic acid, caffeic acid, and ferulic acid in pycnogenol. Therefore, in this study, the reduction in pH values of cooked beef samples treated with different concentrations of EUMFE is beneficial to the preservation of cooked beef.

Color of meat and meat products is an important factor, affecting its marketability and consumer’s purchase preferences (Carpenter et al., 2007). In this study, the addition of EUMFE decreased the L* and b* values, and increased b* and ΔE* values of cooked beef samples which might be due to the red-brown color of the EUMFE. The color of the cooked beef would gradually darken during storage since myoglobin was oxidized to metmyoglobin along with formation of hemichrome in the meat (Verma et al., 2019). Therefore, the change in L* and a* values of cooked beef treated with EUMFE over storage period might be related to retardation of oxidation reaction by EUMFE. In agreement with our results, it has been shown that treatment of cooked beef with cranberry anthocyanin increased ΔE* value as an indicator of total color change (Gong et al., 2021). Moreover, addition of tea catechins, *Perilla frutescens* var. *acuta* water extract and pycnogenol resulted in color changes of cooked beef, and played roles in stability of meat color during the storage period (Mitsumoto et al., 2005; Ahn et al., 2007; Lee C.W. et al., 2015).

Total volatile basic nitrogen is a commonly used parameter to characterize the freshness of meat products and represent the total content of ammonia, dimethylamine, trimethylamine, and other volatile basic nitrogenous compounds (Li et al., 2016). In this study, the addition of EUMFE significantly decreased TVB-N values during storage as compared to control group (0 MIC), showing a protective effect to reduce the deterioration of quality. In general, cooked beef have a shelf life of about 3–7 days under refrigeration, while the TVB-N value of cooked beef treated with 2 MIC on day 9 was still less than 15 mg/kg, indicating that cooked beef was not spoiled. Therefore, the reduction of TVB-N value in cooked beef samples treated with EUMFE should be related to the antibacterial activity of EUMFE. Similar reduction in TVB-N value of minced beef packaged with polylactic acid film containing propolis ethanolic extract and *Ziziphora clinopodioides* essential oil have been reported (Shavisi et al., 2017). Besides, a combined effect of some natural preservatives produced by mixing clove cinnamon extracts with tea polyphenol, chitosan, propolis, nisin, and lysozyme could slow the development of TVB-N in refrigerated beef (Hao et al., 2013).

Thiobarbituric acid reactive substances values are, generally, considered as an important quality parameter of meat products because they can reflect the degree of lipid peroxidation (Ifesan et al., 2009). Our results showed that the addition of EUMFE can significantly reduce TBARS values in cooked beef as compared to control sample (0 MIC), indicating an inhibition of lipid oxidation. Similarly, Ye et al. (2015) have explained the ability of dihydromyricetin to reduce TBARS in cooked ground beef. As reported by Rojas and Brewer (2010) the grape seed extract reduces the oxidative rancidity and thus improves shelf life of refrigerated cooked beef and pork patties while incorporated to these products at 0.02%. Moreover, previous studies have reported that cooked meat is more sensitive to lipid oxidation than raw meat because of thermal damage in membrane phospholipids along with protein denaturation due to heating (Dai et al., 2014).

## Conclusion

The EUMFE exerts a potential antibacterial effect on *S. aureus* by inducing cell membrane hyperpolarization, decreasing bacterial DNA and protein content, increasing cell membrane permeability, and destroying cell membrane integrity and cell morphology. Moreover, the addition of EUMFE effectively inhibits the growth of *S. aureus*, lowers L*, and b* values, increases a* and ΔE* values, decreases the pH and TVB-N values, and retards the lipid oxidation in cooked beef. Therefore, EUMFE has potential as a food preservative to control *S. aureus* contamination and to improve quality of cooked beef. However, the dose optimization of the natural preservative EUMFE and its application to other food system remains to be further studied.

## Data Availability Statement

The original contributions presented in the study are included in the article/Supplementary Material, further inquiries can be directed to the corresponding authors.

## Author Contributions

PF, MX, HK, and MA conceived and designed the experiments. MX, SL, YY, and YF performed the experiments. PF, LG, and YW supervised the project. MX, YY, PF, and LG analyzed the data. MX, MA, and PF wrote the manuscript. All authors contributed to the article and approved the submitted version.

## Conflict of Interest

The authors declare that the research was conducted in the absence of any commercial or financial relationships that could be construed as a potential conflict of interest.

## Publisher’s Note

All claims expressed in this article are solely those of the authors and do not necessarily represent those of their affiliated organizations, or those of the publisher, the editors and the reviewers. Any product that may be evaluated in this article, or claim that may be made by its manufacturer, is not guaranteed or endorsed by the publisher.

## Supplementary Material

Supplementary Figure 1 The images of **(A)** initial *Eucommia ulmoides* male flowers and **(B)** experimental *Eucommia ulmoides* male flower extract (EUMFE) powders in this study.

Supplementary Figure 2 Growth curves of *Staphylococcus aureus* treated with different concentrations of *Eucommia ulmoides* male flower extract (EUMFE). Each bar represents the mean ± SD of three independent experiments.
