# Supplementary material for: Antibacterial Mode of Eucommia ulmoides Male Flower Extract Against Staphylococcus aureus and Its Application as a Natural Preservative in Cooked Beef
Source: Front Microbiol. 2022 Mar 8;13:846622. doi: 10.3389/fmicb.2022.846622 (PMC8957902; doi:10.3389/fmicb.2022.846622)
Supplement: Supplementary file 1 [file Table_1.doc]

**Supplementary Table 1** Major chemical composition of EUMFE

| Composition | Content | Composition | Content |
| --- | --- | --- | --- |
| Polyphenols | 17.32% | naringenin | 0.74% |
| Total flavonoid | 4.67% | alkaloids | 1.17% |
| Chlorogenic acid | 2.81% | Lutedin | 0.87% |
| Genipin | 0.93% | asperuloside | 0.85% |
| Aucubin | 1.06% | Protein | 29.63% |
| Polysaccharide | 3.28% | Fat | 2.26% |
| Moisture | 5.28% | 3-O-acetylursolic acid acetate | 0.61% |
| Quercetin | 1.24% | daphylloside | 0.92% |
| Pinoresinol diglucoside | 0.79% | Others | 25.57% |
