# Supplementary figures and images for: Antibacterial Mode of Eucommia ulmoides Male Flower Extract Against Staphylococcus aureus and Its Application as a Natural Preservative in Cooked Beef
Source: Front Microbiol. 2022 Mar 8;13:846622. doi: 10.3389/fmicb.2022.846622 (PMC8957902; doi:10.3389/fmicb.2022.846622)

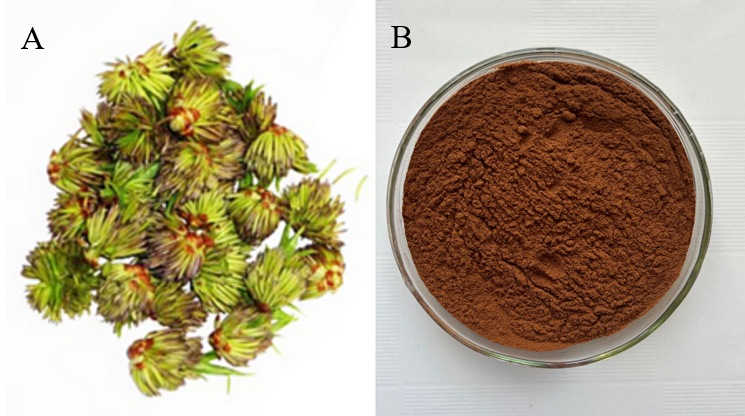

Supplement: Supplementary Figure 1 — The images of (A) initial Eucommia ulmoides male flowers and (B) experimental Eucommia ulmoides male flower extract (EUMFE) powders in this study. [file Image_1.TIF]

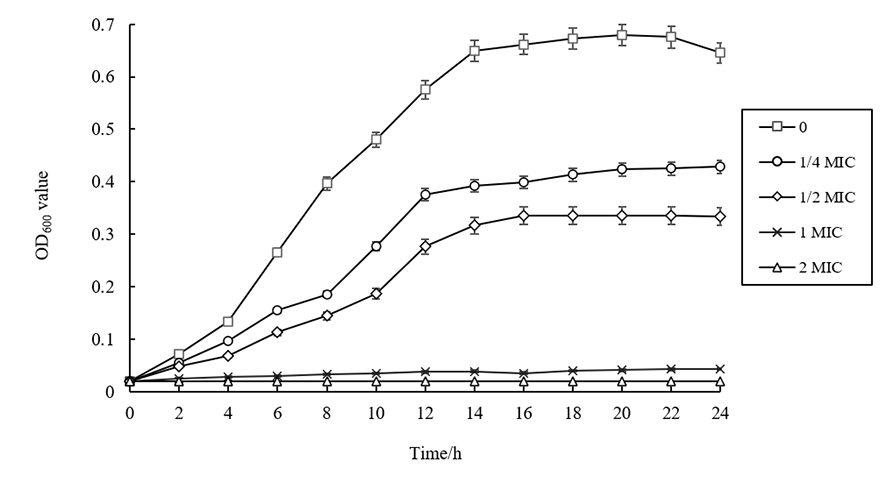

Supplement: Supplementary Figure 2 — Growth curves of Staphylococcus aureus treated with different concentrations of Eucommia ulmoides male flower extract (EUMFE). Each bar represents the mean ± SD of three independent experiments. [file Image_2.TIF]
